# Reversible data hiding techniques with high message embedding capacity in images

**DOI:** 10.1371/journal.pone.0231602

**Published:** 2020-05-29

**Authors:** Furqan Aziz, Taeeb Ahmad, Abdul Haseeb Malik, M. Irfan Uddin, Shafiq Ahmad, Mohamed Sharaf

**Affiliations:** 1 Center of Excellence in IT, Institute of Management Sciences, Peshawar, Pakistan; 2 Centre for Computational Biology, University of Birmingham, Birmingham, England, United Kingdom; 3 Department of Computer Science, University of Peshawar, Peshawar, Pakistan; 4 Institute of Computing, Kohat University of Science and Technology, Kohat, Pakistan; 5 Department of Industrial Engineering, College of Engineering, King Saud University, Riyadh, Kingdom of Saudi Arabia; K L University, INDIA

## Abstract

Reversible Data Hiding (RDH) techniques have gained popularity over the last two decades, where data is embedded in an image in such a way that the original image can be restored. Earlier works on RDH was based on the Image Histogram Modification that uses the peak point to embed data in the image. More recent works focus on the Difference Image Histogram Modification that exploits the fact that the neighbouring pixels of an image are highly correlated and therefore the difference of image makes more space to embed large amount of data. In this paper we propose a framework to increase the embedding capacity of reversible data hiding techniques that use a difference of image to embed data. The main idea is that, instead of taking the difference of the neighboring pixels, we rearrange the columns (or rows) of the image in a way that enhances the smooth regions of an image. Any difference based technique to embed data can then be used in the transformed image. The proposed method is applied on different types of images including textures, patterns and publicly available images. Experimental results demonstrate that the proposed method not only increases the message embedding capacity of a given image by more than 50% but also the visual quality of the marked image containing the message is more than the visual quality obtained by existing state-of-the-art reversible data hiding technique. The proposed technique is also verified by Pixel Difference Histogram (PDH) Stegoanalysis and results demonstrate that marked images generated by proposed method is undetectable by PDH analysis.

## 1 Introduction

Since the beginning of the internet, images or other types of data are constantly shared. For information technology communication, it is an important factor to keep the information secure. Different techniques of cryptography are created to secure the secrecy of communication. But it is not enough that the content of a message are kept secret, it is also necessary to keep existence of the message secret. Steganography [1, 2] is the technique that allows invisible communication i.e. it hides a message inside an object such as image, audio, video etc. Images are the most popular medium of transmission over the internet because of its harmless and attractive nature. In image steganography, data is inserted into an image and a resultant image (or marked image) is produced, which can be easily shared with others. Only the recipient can decode the secret message from the image.

Data hiding techniques enable secret messages to be embedded in images in an undetectable manner, so that the original image and the marked image (image with embedded data) remain visually similar. Any difference in the marked image and the original image is considered as noise and hence the embedded message can not easily be retrieved by any third party. However, the number of bits embedded in an image, also known as embedding capacity, is very low in existing data hiding techniques. Therefore, the embedded message is very short (only few characters). Different data hiding algorithms have been used to improve the embedding capacity of images keeping the Peak Signal to Noise Ratio (PSNR) in acceptable limits. PSNR is used to determine the visual quality of the image. Two main types of data hiding methods are commonly used (i) reversible data hiding and (ii) non-reversible data hiding. In reversible data hiding (RDH) techniques, the original image from the marked image is extracted [3–5] along with embedded data. While in non-reversible data hiding schemes, the original image is permanently distorted and cannot be recovered from marked images [6–8]. RDH algorithms are used in sensitive applications where high precision is required such as, military and intelligence communication, private communication and protecting civilian speeches against opponents. Hence, the extraction of the original image and the visual quality of the marked image are equally important.

Traditional RDH algorithms have used smoother regions of an image to embed data in images [9–11]. In these algorithms the embedding capacity of an image depends on the availability of smooth regions in the image. Images having more smooth regions are capable of embedding large amount of data without any loss in visual quality. However, these algorithms are unable to embed large amount of data in images having non-smooth regions like textures and patterns. Consider, for example, a repeated pattern image where a particular object with high intensity variations appears multiple times in the image. In such cases, the smooth regions are very low. However, if the pixels of the image are rearranged in a different order, we may get more smooth regions in the image.

In this paper, a novel reversible data hiding approach is presented which exploits the presence of patterns and textures in an image. Instead of taking difference between neighboring pixels, the proposed algorithm search for patterns in the image with the possibility of high embedding capacity. The proposed scheme not only increases the embedding capacity of an image, but also aims in increasing the visual quality of the marked image. One of the advantages of the proposed method is that it can provide high embedding capacity in applications, where pattern images are transferred. For example in web pages an image with repeated patterns is generally used as a background image. This image can be used to transfer large amount of data to the client. Another advantage of the proposed method is that it produces a transformed image as an intermediate step which can be used in combination with any other difference based method to increase the embedding capacity. Finally, only a single value *δ* is transferred as overhead information.

The rest of the paper is organized as follows. A detailed literature review is presented in 2. The proposed reversible data hiding technique is explained in 3. Experimental results of the proposed method and its comparisons with existing state-of-the-art methods are described in 4 and the paper is concluded in 5.

## 2 Literature review

Over the last two decades, a number of RDH methods have been proposed based on lossless data compression techniques. These schemes involve methods that apply lossless compression to a selected set of features from the original image and then embed the message in the space that has been saved due to feature compression. One of the earliest works [12], have proposed a data embedding framework having the property that the original image can be fully recovered once the data has been extracted from the marked image. They have presented methods for both the uncompressed formats (BMP, TIFF) and for the JPEG format. Another technique [13] have presented a lossless generalized LSB embedding scheme (G-LSB) which is based on grouping the pixels of an image and embedding data bits into the state of each group. To increase the embedding capacity of RDH, a different scheme called Difference Expansion (DE) has been proposed [14]. The scheme is based on modifying the difference between a pair of pixel values while keeping the average of them unchanged. DE achieves good performance since natural images, in general, exhibiting high correlation between adjacent pixels. In [15], novel methods were proposed to resolve two issues associated with DE method, i.e. the maximum number of embeddable location and the payload control capability.

Another important category of RDH algorithms is based on modifying the histogram of the grey intensities of the original image. The first histogram-based method was proposed in [4], where the peak point and the zero point of image histogram are used to embed the data. The histogram of the image is computed and the peak point ‘P’ and the closest zero point (to the peak point) ‘Z’ are determined. The histogram from the peak point is then shifted towards zero point thereby creating a capacity equal to the peak point for the data to be embedded. To embed the data, the modified image is scanned. Once a pixel with value ‘P’ is encountered, the next bit value of the message is added to the pixel value. In this method, since each pixel value is incremented by 1 at most, the visual quality of the marked image is generally very high. However, the method achieves a very low hiding capacity. For example, the largest embedding capacity was only 5kb when Lena’s image was used as a test image. The technique also fails when an image has uniform histogram.

To overcome the limitations associated with histogram-based method, a number of extensions have been proposed over the recent years [16–18]. The main objective is to increase the embedding capacity of the histogram-based methods. Most of these techniques consider the histogram of the difference image (i.e. taking the differences between adjacent pixels) instead of the original image. This is because of the fact that neighbouring pixels are strongly correlated and the difference is expected to be very close to zero for most of the pixels. This can cause a prominent maximum in the histogram of the difference image, which can ultimately result in high data embedding capacity. A reversible image authentication technique based on difference image have been proposed in [16]. First, a difference image is computed by taking the difference of grey level values in each pair of pixels. The difference image has half the size of the original image. Then data is embedded in the difference image and the marked image is constructed using modified difference image. A multilevel hiding strategy that can achieve a large hiding capacity have been proposed in [9]. An auxiliary binary tree that predetermine multiple peak points in the difference histogram, to solve the issue of communication of multiple peak points have been proposed in [19].

A high-fidelity reversible data hiding scheme for images which is based on a prediction strategy and prediction-error expansion (PEE) technique have been proposed in [20], where a new prediction strategy have been introduced, referred to as Pixel-Value-Ordering (PVO). In PVO, data is first divided into non-overlapping equal-sized blocks. Then the pixels in each blocks are sorted and the maximum and minimum values of each block are predicted by other pixels of the block according to their pixel value orders. The PVO based PEE method has an advantage that, it not only improves the embedding capacity of the image but also alleviate the degradation in image quality. A number of extensions have been proposed in PVO based method. An RDH approach based on invariant pixel-value-ordering is proposed in [21], where instead of considering only a single pixel with maximum/minimum value of a block, all the maximum/minimum-valued pixels are considered as a unit to embed data. In [22], a Pixel-based PVO (PPVO) is used to improve the embedding capacity of original PVO-based method by considering each pixel using its sorted context pixel. A modification to PPVO [23] have further enhanced the embedding capacity. Another attempt to improve the performance [24] have introduced IPVO, that utilizes a new approach for computing differences. Some other details about various reversible data hiding schemes based on pixel value ordering can be found in [25–28]. More related studies can be found at [29–31].

In [1], differencing and substitution approaches are used by dividing image into 3 × 3 non-overlapping blocks and then applied Least Significant Bit (LSB) substitution on first two LSBs and Quotient Value Differencing (QVD) on remaining six bits. They proved that Pixel Difference Histogram (PDH) and RS analysis technique cannot detect the proposed steganography mechanism and efficiently improved the embedding capacity. In [32], authors proposed a technique by taking difference of overlapped and modulus function of overlapped pixel difference. The quality and embedding capacity has been improved. The security of the method has also been verified by RS analysis. In [33], authors have proposed PVD (Pixel-Value Differencing) technique by improving the embedding capacity and PSNR value. The method is also proved to resist from PDH and RS analysis. Authors introduced a novel PVD approach in [34] to further improve the image quality and hiding capacity. The scheme has been experimentally verified against the resistant of RS attack. In [35], blocked base bit flipping technique is introduced. In the paper, 7^th^ and 8^th^ LSB is used for location map. The method gives high embedding capacity and quality index is improved from state-of-the-art bit flipping techniques. In [36], authors have introduced a new LSB based technique by exploiting the edges of block in eight directions which possesses higher embedding capacity. In [37], the proposed method addressed three problems i.e. fall off boundary problem, pixel difference histogram and RS analysis. Authors in [37] and [38] combined PVD and LSB substitution techniques which improved the capacity and lesser distortion. In [38], two approaches are introduced i.e. replacement of n-rightmost bit and modified pixel value differencing. The technique not only improved the PSNR value and embedding capacity but also ensured high fidelity to salt and pepper noise, RS analysis and PDH analysis. Another approach introduced in [39], where experimental investigation was carried out that the proposed technique is resistant to steganalytic attacks. In [40], improved LSB matching, such as dual stego-image based pixel pair and dual stego-image based modified LSB is introduced. In [2], dual-layered based RDH method using modified LSB matching has been proposed by enhancing the embedding capacity and curtail the distortion by verifying to resist RS and PDH analysis. In [41], authors have proposed a new DE (Difference Expansion) embedding algorithm, which utilizes the horizontal as well as vertical difference images for data hiding.

All techniques presented in this section have used different techniques to improve the embedding capacity of an image to hold a secret message and at the same time increase PSNR value so that a naked eye cannot notice any changes in the image. The best results are achieved by multilevel histogram modification of difference images by Lin et. al. [9]. The proposed technique presented in this paper further improves the performance as demonstrated in section 4. To the best of our knowledge, none of the research articles have studied the problem of increasing the embedding capacity and PSNR from the same perspective as studied in this paper.

## 3 Proposed reversible data hiding technique

Existing data hiding techniques, such as *difference expansion* [14] and histogram modification of difference images [9], are based on detecting smooth regions to embed data. The higher the number of smooth regions, the higher the embedding capacity of a message in the image. In this paper, we are proposing a reversible data hiding technique that exploits the presence of repeating patterns and texture in images. In this method the input image is transformed by shifting rows or columns to maximize the embedding capacity. At the sender side the data/message is embedded in the image to hide the data. Then at the receiving end, the extraction of the data is performed from the image and the original image is restored. The effectiveness of any RDH scheme is measured by the following three properties:

- Embedding capacity: The maximum amount of data that can be embedded in the image, but able to recover the original image at the same time.
- Visual Quality: The visual quality of the marked image and its comparison with the original image.
- Complexity: The complexity of algorithms for embedding and extracting data.

The proposed algorithm consists of two steps:

1. Data embedding in the input image
2. Data extraction from marked image

### 3.1 Data embedding in the input image

Suppose we have a grey-scale image of size *width* × *height*. The number of steps required to embed a message in the image is given below:

- Step 1: Generate index map
  For all values of *δ* from 1 to *width* − 1, compute an index map and its embedding capacity. Select the value of *δ* to be the one that has the maximum capacity, and keep a copy of the corresponding index map.
- Step 2: Image transformation by index map
  The input image is transformed using the index map that has the maximum capacity i.e. the columns in the input image are switched using the index map. The resultant image has different columns ordering than the input image.
- Step 3: Difference image with embedded data
  The difference matrix is calculated for the transformed image using the index map. The peak intensity in the difference image is used to shift the histogram to create space for the message to be embedded i.e. all values greater than the peak value are incremented by 1. The message is then embedded in the difference image with histogram shifting by scanning for all 1 in the image and message. The size of the secret message depends upon the maximum capacity. We also keep track of the value of *δ* and whether a row or column wise transformation was used.
- Step 4: Transformed image to marked image
  The transformed image is embedded with data using the difference image with embedded data and finally using inverse-map of the index map to rearrange the rows or columns of the transformed image. The output will be actually the original image with embedded message, known as marked image.

**Fig 1:**
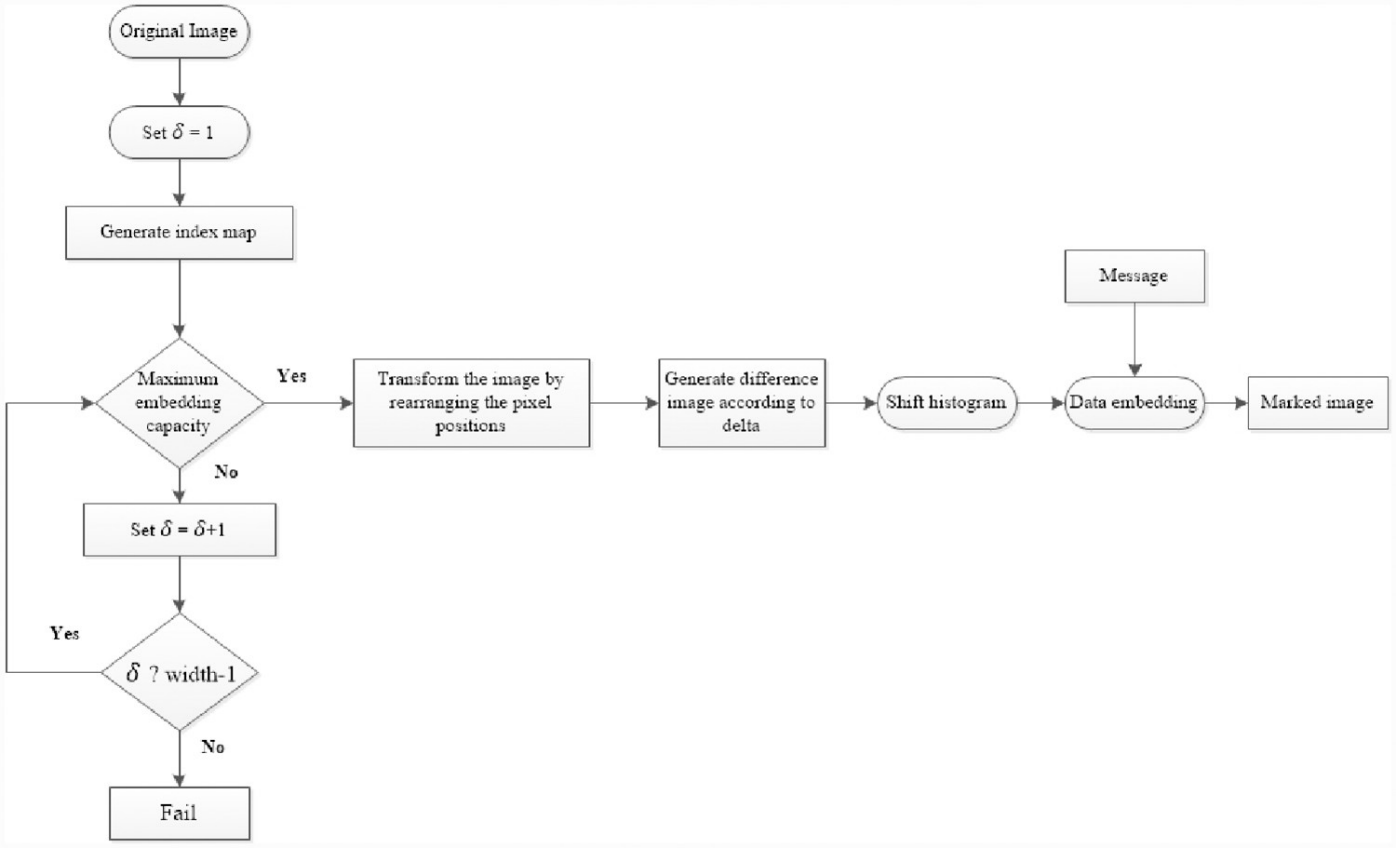
Flowchart of all steps required for the data embedding in images.

The flow chart of the data embedding in image is given in Fig 1. A detailed explanation of all steps with concrete examples is given below.

#### Step 1: Generate index map

In difference based methods [9, 16] uniform regions of an image to embed data are exploited. In those methods, the value of a pixel is increases by at most 1. This guarantees that the visual quality of the image is not significantly reduced. However, in pattern images, the number of uniform regions are low and therefore there is low embedding capacity. The method proposed in this paper is based on histogram shifting of difference image. Rather than calculating a difference image of adjacent pixels as in difference methods, we compute the difference image of pixels which are *δ* columns/rows away. This method guarantees a higher capacity in embedding because of larger peak compared to difference methods. The algorithm of generating index map for an image for each value of *δ* from 1 to *width* − 1 is shown in Algorithm 1. The main idea behind algorithm 1 is to generate a pixel map that is later used to rearrange the pixels of the image in order to increase the image capacity of the image. Here the value of *δ* is used to determine the gap between the similar pixels of the patterns. In other words, the value of *δ* may be considered as the size of the pattern. Once the value of *δ* is determined, the rows/coloums of the image are rearranged according to the value of *δ*, so that the similar pixels of the patterns (with same intensity) become adjacent to each other. For each index map the embedding capacities are calculated and the index map with the maximum embedding capacity is selected for image transformation.

**Algorithm 1**: Generate Index Map

**Input**: *δ* and *n*          // length of the image array and *δ*

**Output**: *map*                  //Set of indices

**1** Create integer array *map*[1…*n*]

**2** Create boolean array *flag*[1…*n*]

**3** *map*(1) ← 1

**4** *flag*(1) ← true

**5** **for** *i* ← 2 to *n* **do**

**6**  *map*(*i*) ← (*map*(*i* − 1) + *δ*) mod *n*

**7**  **if** *flag*(*map*(*i*)) == true **then**

**8**   *map*(*i*) ← *map*(*i*) + 1

**9**  *map*(*i*) ← true

**Fig 2:**
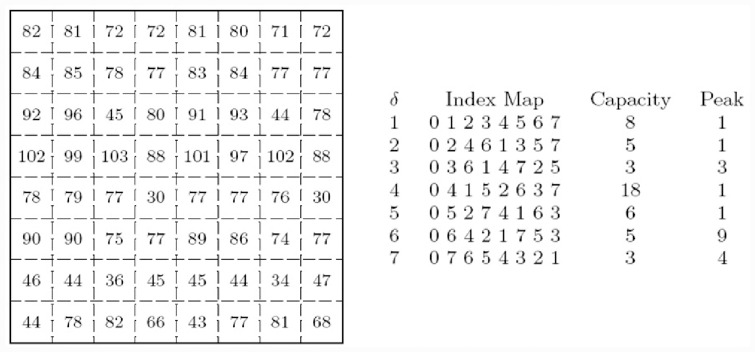
The index map, capacity and peak are computed for different values of *δ* using the Generate Index Map algorithm given in Algorithm 1 for an 8 × 8 gray-scale pattern image.

The demonstration of the algorithm for generating an index map in an 8 × 8 grey-scale pattern image is shown in Fig 2. The algorithm generates the index map for different values of *δ*. The embedding capacity and peak are computing by reordering the columns in the image based on the index map. The maximum capacity in the image is demonstrated when *δ* is equal to fourth column reordering. Therefore, the index map is {0 4 1 5 2 6 3 7}, *δ* is 4, peak is 1 and maximum embedding capacity is 18.

#### Step 2: Image transformation by index map

The image is transformed by rearranging the columns using the index map with the highest embedding capacity. An example is shown in Table 1, where an input image is transformed by switching the columns in the image using the index map = {0 4 1 5 2 6 3 7}.

**Table 1:** Image transformation calculated by switching the columns according to the index map having highest embedding capacity.

82	81	72	72	81	80	71	72
84	85	78	77	83	84	77	77
92	96	45	80	91	93	44	78
102	99	103	88	101	97	102	88
78	79	77	30	77	77	76	30
90	90	75	77	89	86	74	77
46	44	36	45	45	44	34	47
44	78	82	66	43	77	81	68
(a) Original input image.							
82	81	81	80	72	71	72	72
84	83	85	84	78	77	77	77
92	91	96	93	45	44	80	78
102	101	99	97	103	102	88	88
78	77	79	77	77	76	30	30
90	89	90	86	75	74	77	77
46	45	44	44	36	34	45	47
44	43	78	77	82	81	66	68
(b) Transformed image by index map {0 4 1 5 2 6 3 7}.							

#### Step 3: Difference image with embedded data

The difference image in the transformed image is calculated by taking the difference between adjacent columns, rather than taking difference throughout the image. The advantage is that, we have half the number of pixels of the transformed image. The difference image of the transformed input image given in Table 1b is shown in Table 2a. The peak is calculated for the histogram of the difference image. As shown in Fig 2, the peak for the index map {0 4 1 5 2 6 3 7} is 1 and capacity is 18. Which means that we have a maximum of eighteen 1s in the image.

**Table 2:** Difference image, histogram shifting and embedding data in the difference image.

1	1	1	0	1	1	1	0	2	2	2	0
1	1	1	0	1	1	1	0	2	1	1	0
1	3	1	2	1	4	1	3	2	4	2	3
1	2	1	0	1	3	1	0	2	3	2	0
1	2	1	0	1	3	1	0	2	3	2	0
1	4	1	0	1	5	1	0	2	5	2	0
1	0	2	2	1	0	3	3	2	0	3	3
1	1	1	2	1	1	1	3	1	2	1	3
(a) Difference image for image in Table 1b.				(b) Difference image after histogram shifting.				(c) Difference image with data embedded.			

To create space for data/message to be embedded in the input image, the histogram of the difference image is shifted, i.e. all intensities of the difference image greater than the peak value are incremented. After shifting the histogram of the difference image, the data can be embedded. The maximum size of the data/message depends on the maximum capacity. In the example, peak is 1 with embedding capacity as 18. The histogram shifting applied to the difference image is shown in Table 2b.

Since the maximum embedding capacity in the image is 18, we create an example 18-bit pattern 111100111111111010 as data to be embedded in the image. The pattern has 0s and 1s. We scan the difference image with histogram shifting and the data bits from left to right and when there is a 1 in difference image and data we increment the 1 in the difference image by 1. We do not change 1 in the difference image when there is a 0 in the data. We only scan for 1, as the peak for the index map is 1. The embedding of the data in the image with histogram shifting is shown in Table 2c.

#### Step 4: Transformed image to marked image

Using the difference image with embedded data and the index map, the transformed image with data embedded is generated. The difference image of the transformed image with embedded data is visually the same as the difference image with embedded data. Finally an inverse transform is taken based on the index map to calculate the marked image i.e. the order of columns are changed in reverse order to produce the original image with data embedded. For the transform image given in Table 1b, the transformed image with embedded data is shown in Table 3a and the marked image is shown in Table 3b.

**Table 3:** Transformed image with embedded data and the production of the marked image using inverse-transform through index map.

83	81	82	80	73	71	72	72
85	83	85	84	78	77	77	77
93	91	97	93	46	44	81	78
103	101	100	97	104	102	88	88
79	77	80	77	78	76	30	30
91	89	91	86	76	74	77	77
47	45	44	44	37	34	50	47
44	43	79	77	82	81	71	68
(a) Transformed image with embedded data.							
83	82	73	72	81	80	71	72
85	85	78	77	83	84	77	77
93	97	46	81	91	93	44	78
103	100	104	88	101	97	102	88
79	80	78	30	77	77	76	30
91	91	76	77	89	86	74	77
47	44	37	50	45	44	34	47
44	79	82	71	43	77	81	68
(b) Marked image, where the data is embedded in the input image.							

### 3.2 Data extraction from marked image

**Fig 3:**
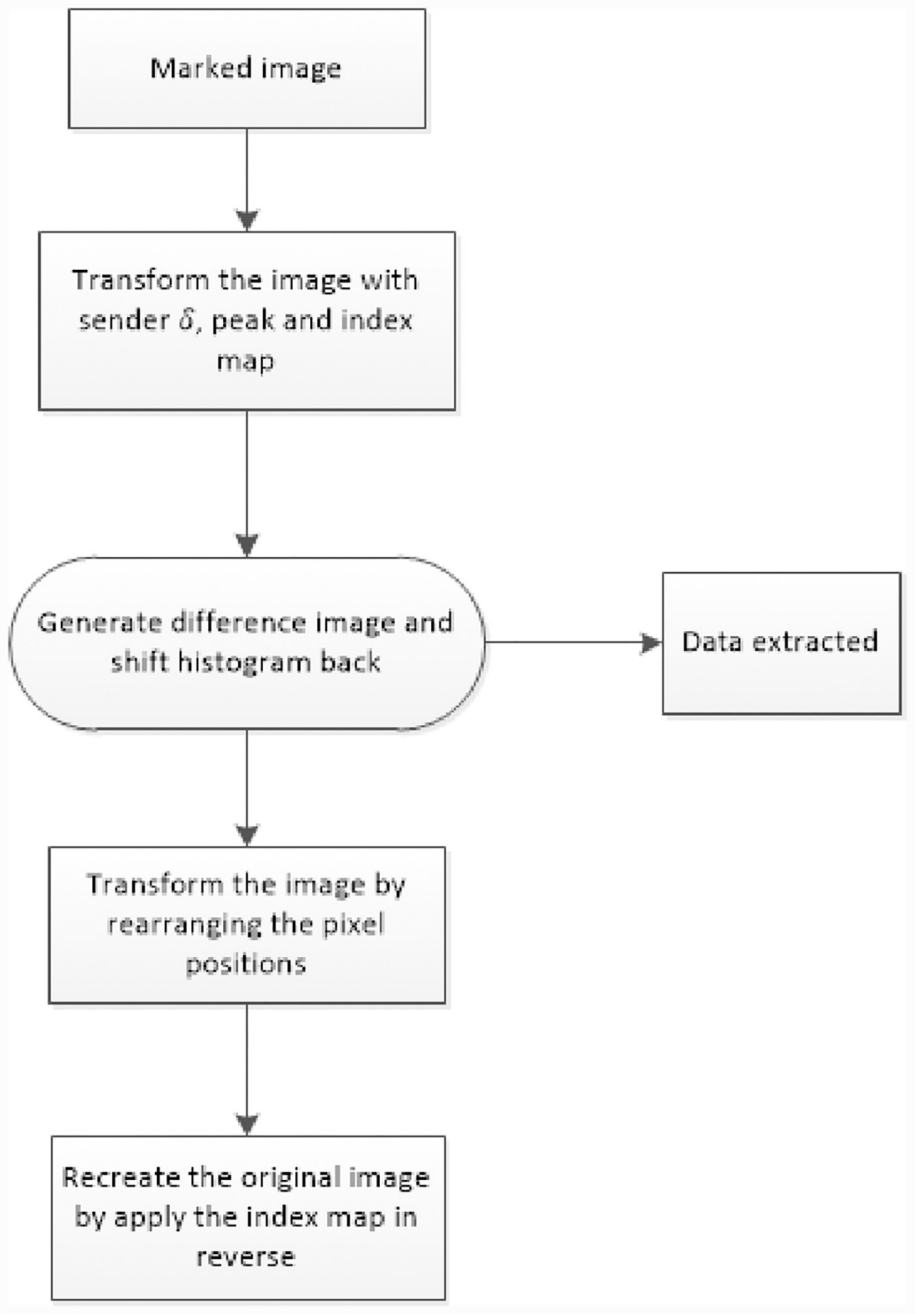
Flowchart explaining all steps in data extraction from marked image.

The sender has embedded a secret message in the input image and sent it as a marked image. Now at the receiving end, we would like to extract the message from the image along with the original image. To recover message and original image, we need the values of *δ*, index map and peak along with the marked image. The data extraction process from marked image is given in Fig 3. The number of steps required to extract the secret message and the original image is given below.

- Step 1: Marked image to transformed image
  Using the *δ* value and index map used by the sender to embed the data in the image, we rearrange the rows or columns of the marked image to generate the transformed image.
- Step 2: Difference image and histogram shifting in reverse
  The difference image is calculated for the transformed image. From the difference image we extract data using the peak intensity, leaving behind the shifted difference of the host image. Then we apply histogram shifting in reverse for all values greater than the peak value.
- Step 3: Difference matrix to compute the transformed image
  Using the difference image with histogram shifted in reverse, we generate the transformed image.
- Step 4: Recreate the original image
  Finally, using index map in reverse, rearrange the rows or columns of the transformed image. This will produce original image.

All the steps required to extract the data and the original image are explained in detail along with examples are given below.

#### Step 1: Marked image to transformed image

The example marked image received by the receiver is shown in Table 4a, where we apply the index map = {0 4 1 5 2 6 3 7} to generate the transformed marked image shown in Table 4b.

**Table 4:** Converting marked image to transformed image.

83	82	73	72	81	80	71	72
85	85	78	77	83	84	77	77
93	97	46	81	91	93	44	78
103	100	104	88	101	97	102	88
79	80	78	30	77	77	76	30
91	91	76	77	89	86	74	77
47	44	37	50	45	44	34	47
44	79	82	71	43	77	81	68
(a) Marked image.							
83	81	82	80	73	71	72	72
85	83	85	84	78	77	77	77
93	91	97	93	46	44	81	78
103	101	100	97	104	102	88	88
79	77	80	77	78	76	30	30
91	89	91	86	76	74	77	77
47	45	44	44	37	34	50	47
44	43	79	77	82	81	71	68
(b) Transformed image using the index map = {0 4 1 5 2 6 3 7}.							

#### Step 2: Difference image and histogram shifting in reverse

We compute the difference image of the transformed image. The difference image with data embedded for the transformed image given in Table 4b is computed in Table 5a.

**Table 5:** Difference image with data, data extraction by peak values and histogram shifting in reverse.

2	2	2	0	1	1	1	0	1	1	1	0
2	1	1	0	1	1	1	0	1	1	1	0
2	4	2	3	1	4	1	3	1	3	1	2
2	3	2	0	1	3	1	0	1	2	1	0
2	3	2	0	1	3	1	0	1	2	1	0
2	5	2	0	1	5	1	0	1	4	1	0
2	0	3	3	1	0	3	3	1	0	2	2
1	2	1	3	1	1	1	3	1	1	1	2
(a) Difference image with data.				(b) After data extraction by peak value.				(c) Histogram shifting in reverse.			

Since the peak value is 1 and in the data embedding process, we have incremented 1 in the difference image by 1 if there is a 1 in the bit pattern of data. Therefore to extract the data from the image we scan the difference image with data from left to right and every time we encounter a 2 we store 1 in our data stream and decrement 2 by 1 in the difference image. If we encounter a 1 in the difference image, we store 0 in the data bits. This data extraction by peak value 1 is shown in Table 5b.

At the sender side, histogram shifting was applied to increment all values greater than the peak value. In the reverse process, we decrement all values greater than the peak value by 1. This process is shown in Table 5c.

#### Step 3: Difference matrix to compute the transformed image

The difference matrix with histogram shifted in reverse is used to compute the transformed image as shown in Table 6a.

**Table 6:** Transformed image to original input image.

82	81	81	80	72	71	72	72
84	83	85	84	78	77	77	77
92	91	96	93	45	44	80	78
102	101	99	97	103	102	88	88
78	77	79	77	77	76	30	30
90	89	90	86	75	74	77	77
46	45	44	44	36	34	45	47
44	43	78	77	82	81	66	68
(a) Recreating transformed image.							
82	81	72	72	81	80	71	72
84	85	78	77	83	84	77	77
92	96	45	80	91	93	44	78
102	99	103	88	101	97	102	88
78	79	77	30	77	77	76	30
90	90	75	77	89	86	74	77
46	44	36	45	45	44	34	47
44	78	82	66	43	77	81	68
(b) Recovered original input image.							

#### Step 4: Recreate the original image

The original image is recovered from the transformed image, by applying the index map in reverse. This process is shown in Table 6b.

Note that the value of the delta can be easily communicated by embedding it with the secret message. This will require us to sent additional one byte (or two in case of large images) of data along with the message. In our approach, we have reserved the first two bytes for the value of *δ*. In this case we need to embed two bytes of information (for image of size less than or equal to 256, only one byte is required). The algorithm is published on dx.doi.org/10.17504/protocols.io.bdyzi7x6 for online visibility.

### 3.3 Computational complexity

In this section we give computational complexity of the proposed framework. Note that all the calculations in our proposed algorithm are based on spatial domain, which means that the running time of the framework is based on the number of pixels in the image. Assume that the size of the image is *M* × *N*, i.e., the height of the image is *M* while its width is *N*. The hiding phase of the algorithm requires *O*(*MN*) time as it scans the whole image once. To determine the optimal value of *δ*, we need to repeat this process *N* + *M* times. Therefore the running time of the embedding procedure is *O*((*M* + *N*) × *MN*)). Assuming *M* = *N* = *n*, the running time becomes *O*(*n*^3^). The extraction time, however, does not significantly change as we already know the value of *δ*.

## 4 Results

The proposed technique of reversible data hiding is evaluated by embedding data in different standard images. The results are compared with existing difference methods techniques to evaluate the maximum capacity and PSNR for image visual quality.

**Fig 4:**
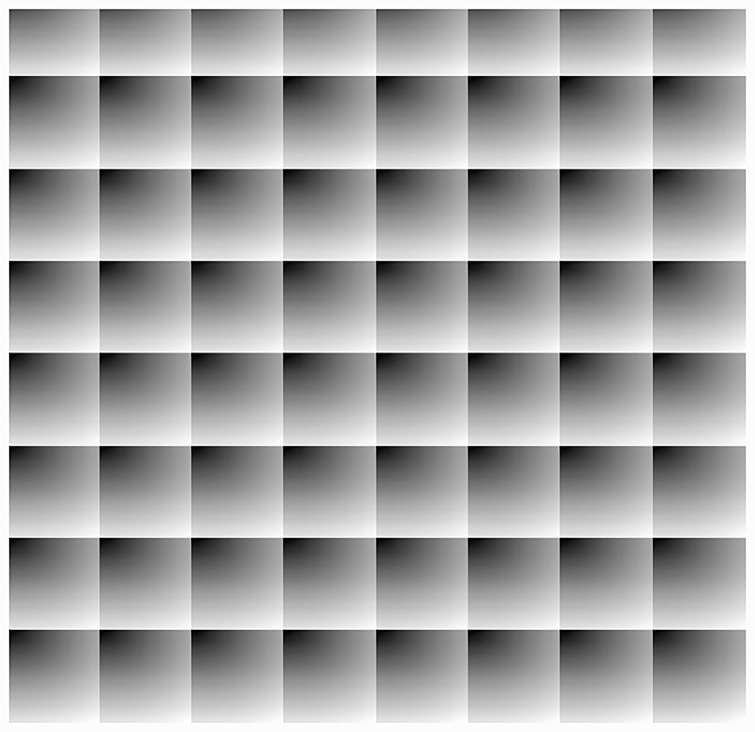
A 512 × 512 checkerboard.

**Fig 5:**
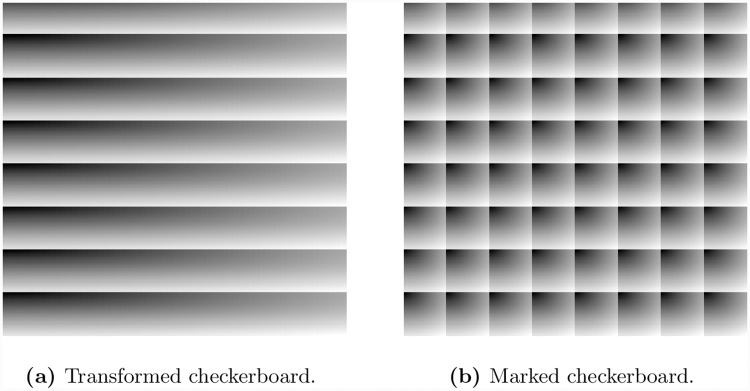
Embedding of data in checkerboard image.

We take an example of a checkerboard shown in Fig 4. This 512 × 512 image is generated in Matlab and has patterns that are repeating after every 64 pixels (both horizontally and vertically). In order to compute the value of *δ* for the image, we have applied Algorithm 1 on the image and have computed an index map. Next, to embed data in the image, we use the generated index map to transform the image. The resultant image is shown in Fig 5a. Note that pixels with similar intensities appear close to each other in the transformed image, resulting in more smooth regions in the image and, hence, higher embedding capacity (i.e. 131, 072 in proposed method compared to 54, 342 in existing multilevel histogram modification of difference images by Lin et. al [9]), which means more than 100% improvement. c.f. Table 7). We generate a payload message of size equal to the embedding capacity and embed it in the transformed image. Finally, we apply the inverse mapping and recover the original image with embedded data as shown in Fig 5b.

**Table 7:** Comparison of maximum capacity and PSNR in different images achieved by proposed embedding and other state-of-the-art techniques. (Cap. means embedding capacity in bits).

	Proposed		Method in [9]		Method in [43]		Method [44]	
Image	Cap.	PSNR	Cap.	PSNR	Cap.	PSNR	Cap.	PSNR
Checkerboard	131072	54.1400	54342	53.5262	50382	51.2827	50121	51.2828
Large pattern	80948	52.7678	51828	52.1102	51020	51.2029	50202	51.2202
Small patterns	46165	54.1257	27700	53.5500	26292	52.2322	26202	52.2302
Bubbles	48029	53.4189	41672	53.6853	27292	51.2278	26302	51.2190
House	21527	51.5442	20667	51.5298	14310	48.2000	14231	48.0683
Butterfly	35271	51.7676	30660	51.6808	7455	51.7332	6071	51.4336
Texture 1	14195	51.3988	6195	51.2673	6101	51.2239	6010	51.2100
Texture 2	15330	51.4103	5132	51.2425	5100	51.1891	5090	51.1090
Clown	55663	52.1752	42855	51.9177	4606	42.1829	5200	52.1500
Ship	25186	52.1216	22481	51.9892	7301	48.2000	14181	51.6528
Barbara	18466	51.8276	15819	51.7118	2217	53.7827	1566	51.6301
Zelda	26222	52.1334	19896	51.8634	2588	50.7940	19896	51.8620

**Fig 6:**
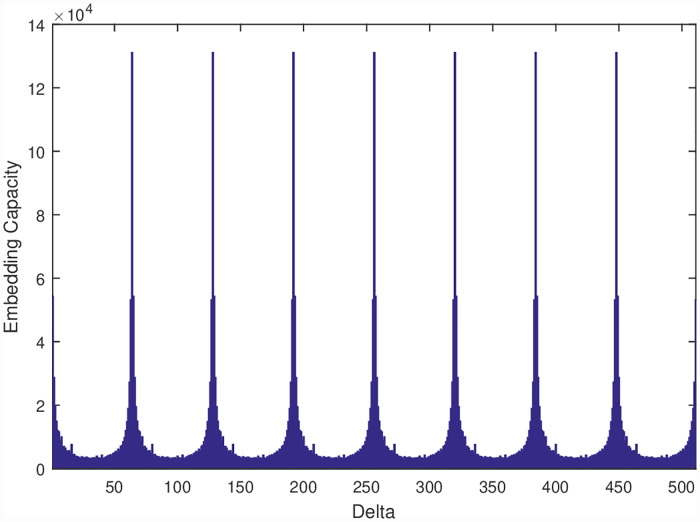
Embedding capacity for different values of *δ* for checkerboard. As noted we have high capacity when we have *δ* in multiple of 64.

Note that since the image has small repeating patterns, we can achieve the same embedding capacity with different values of *δ*. To demonstrate, we have plotted the embedding capacities against the values of *δ* in Fig 6 showing that when the value of *δ* becomes close to 64, the embedding capacity increases up to 120k. It is also important to note that we get multiple peaks for this image. This is due to the fact that the same pattern is repeating after every 64 pixels. For this reason, we get a peak for all values of *δ* < 512, that are multiples of 64. In our experiments, we select the smallest value of *δ* (64 in this case).

**Fig 7:**
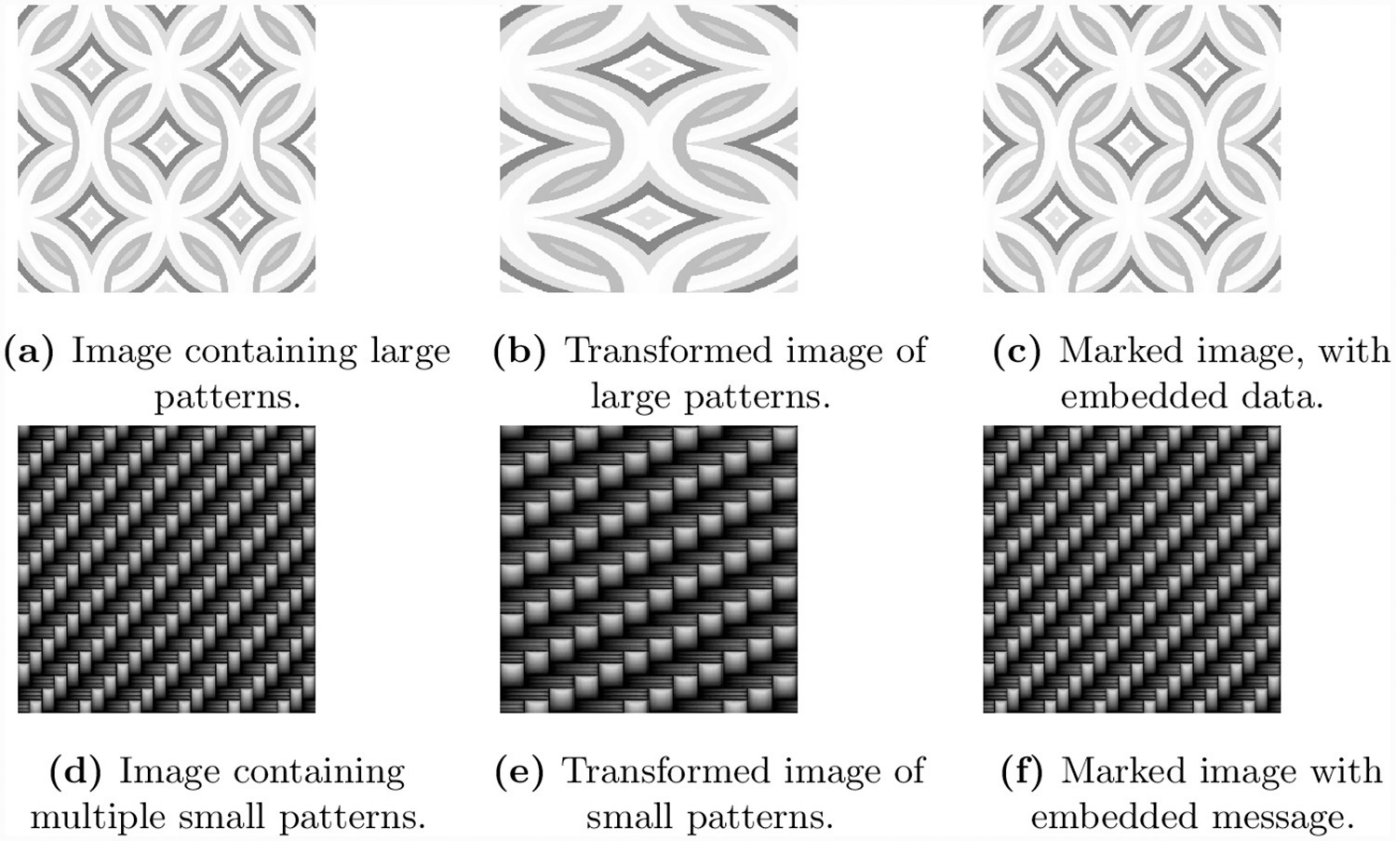
Image with multiple repeated large and small patterns.

To further investigate the performance of the proposed algorithm, we select two more images of patterns each of size 512 × 512. The first image has one large pattern with size 256 × 256 shown in Fig 7a, while the second image has multiple small patterns shown in Fig 7d. In order to find the embedding capacity, we have executed Algorithm 1 on each of these images and computed the value of *δ* as 256 that gives maximum embedding capacity. Next we have transformed the image using the index map generated by the algorithm. The transformed image for the large patterns is shown in Fig 7b. Note that our algorithm has merged the two patterns in the input image into a single pattern. A similar transformation for the image with small repeated patterns is shown in Fig 7e. Once the image has been transformed and maximum capacity has been determined, we have embedded a message in the transformed image and have applied inverse mapping to get the marked image shown in Fig 7c and 7f.

In order to compare the performance of the proposed method, we have compared it with the difference method that does not apply any transformation on the image. For this purpose, we have computed the Peak Signal to Noise Ratio (PSNR) for both methods. PSNR is generally defined in terms of the Mean Squared Error (MSE). The MSE of two images of sizes *m* × *n* is defined as

$$\begin{array}{c} MSE=\frac{1}{mn}\sum_{i=0}^{m-1}\sum_{i=0}^{n-1}{\left[I(i,j)-K(i,j)\right]}^{2}, \end{array}$$

where the image *K* is considered as a noisy approximation of the image *I*. The PSNR is then defined as

$$\begin{array}{c} PSNR=10\times \log(\frac{255^{2}}{MSE}). \end{array}$$

**Fig 8:**
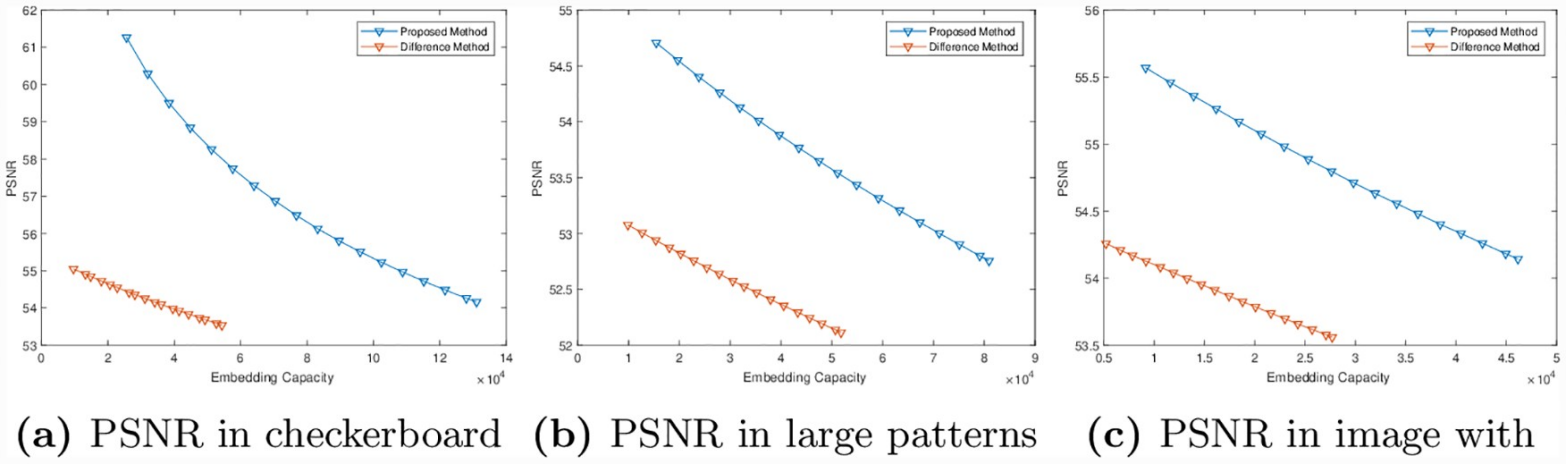
Performance comparisons between the proposed method and difference method for images given in Figs 5b, 7c and 7f respectively.

The value of PSNR against the embedding capacity for the three images shown in Figs 5b, 7c and 7f is plotted in Fig 8. The figure shows the comparison of the proposed method with difference method and demonstrates that the embedding capacity for the three images has increased, while the PSNR value has also been improved. The difference is significant in the case of checkerboard image.

**Fig 9:**
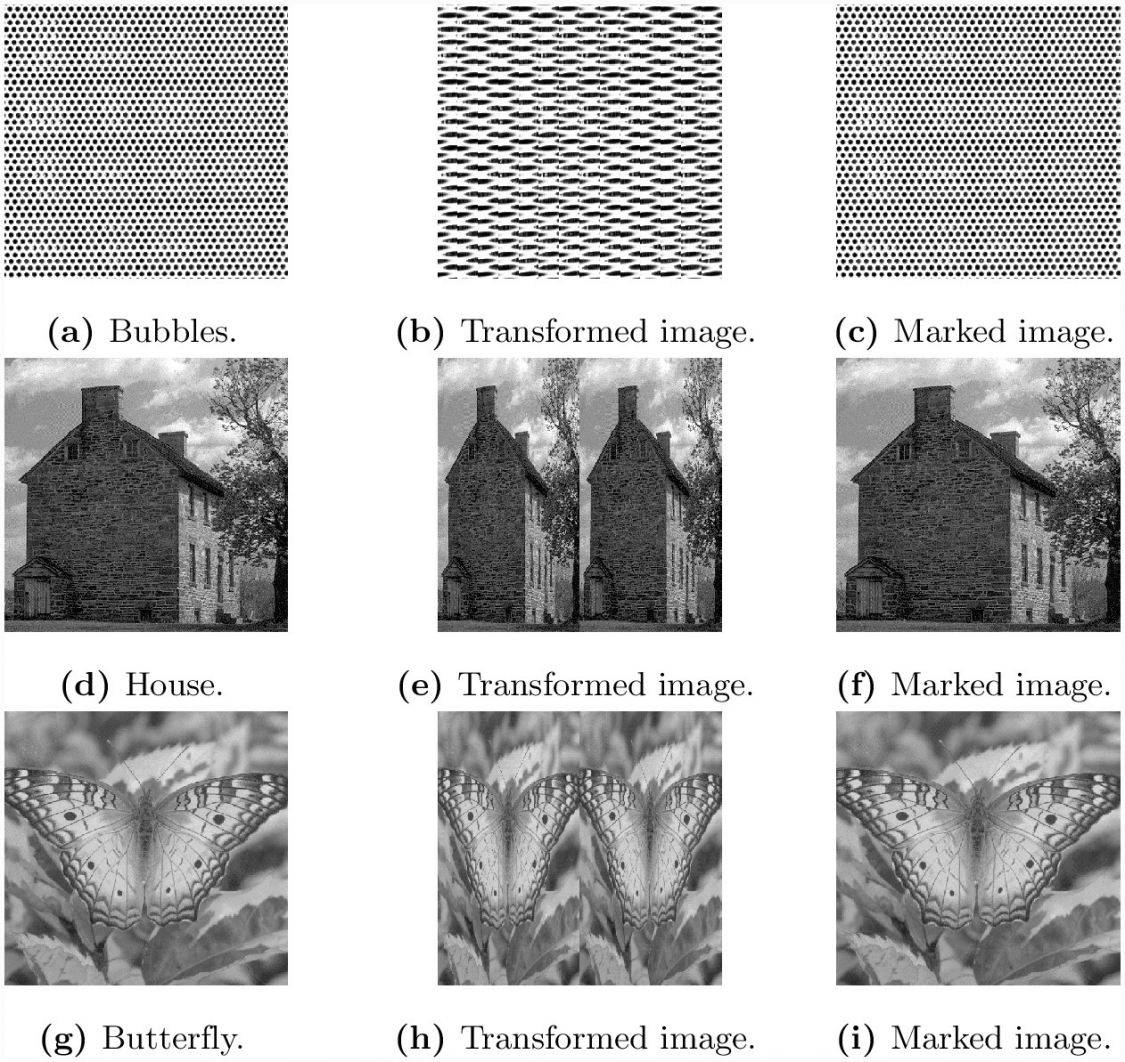
Data embedding in three publicly available images.

**Fig 10:**
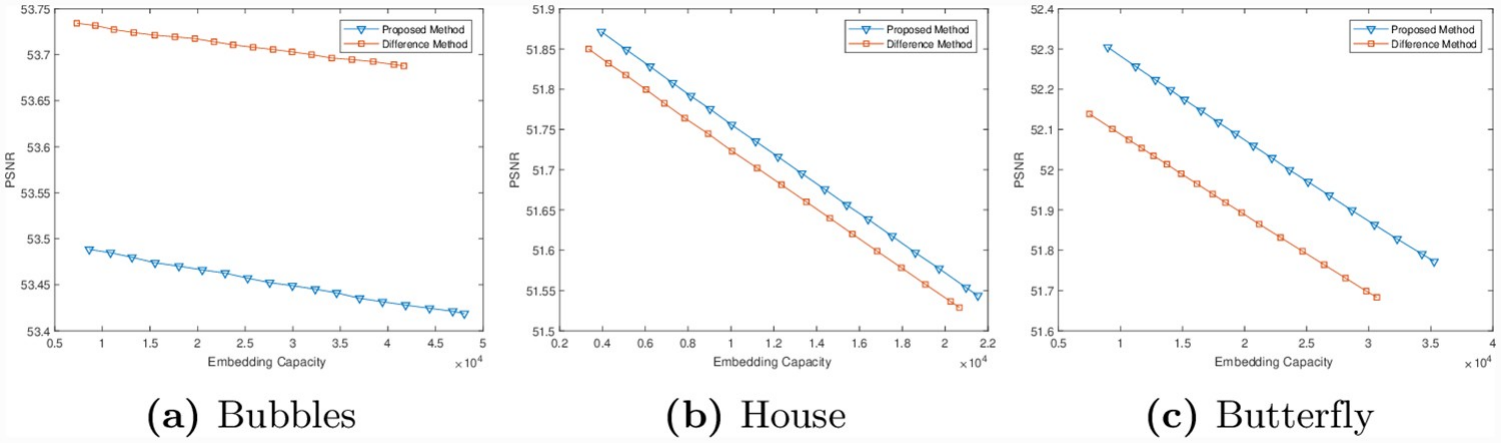
Performance comparison of embedding techniques with difference method of three publicly available images.

To further investigate the performance of the proposed method we have tested it on three publicly available images. These three images are shown in Fig 9a, 9d and 9g respectively. For each of these images, we have computed the value of *δ* and have constructed transformed images. The corresponding transformed images in each case is shown in the middle column of Fig 9. Note that in the first image, a pattern (circle) is clearly visible and the transformed image was constructed by combining these patterns. In the other two cases, the transformed image was constructed by taking the alternate columns of the image. Next, we have embedded data in the transformed images to produce marked images. The right most column of Fig 9 shows the marked images with embedded data. The visual quality is not affected, which means no body can even have a slight idea that the image has embedded data. In Fig 10, we have plotted the data capacity against the PSNR values for the three images i.e. Bubbles, House and Butterfly obtained by the proposed method and difference method. Note that in all cases, except for the first image, we have achieved higher PSNR values compared to existing difference method. Although the PSNR value for the proposed method is low in the first case, the difference is not significant.

**Fig 11:**
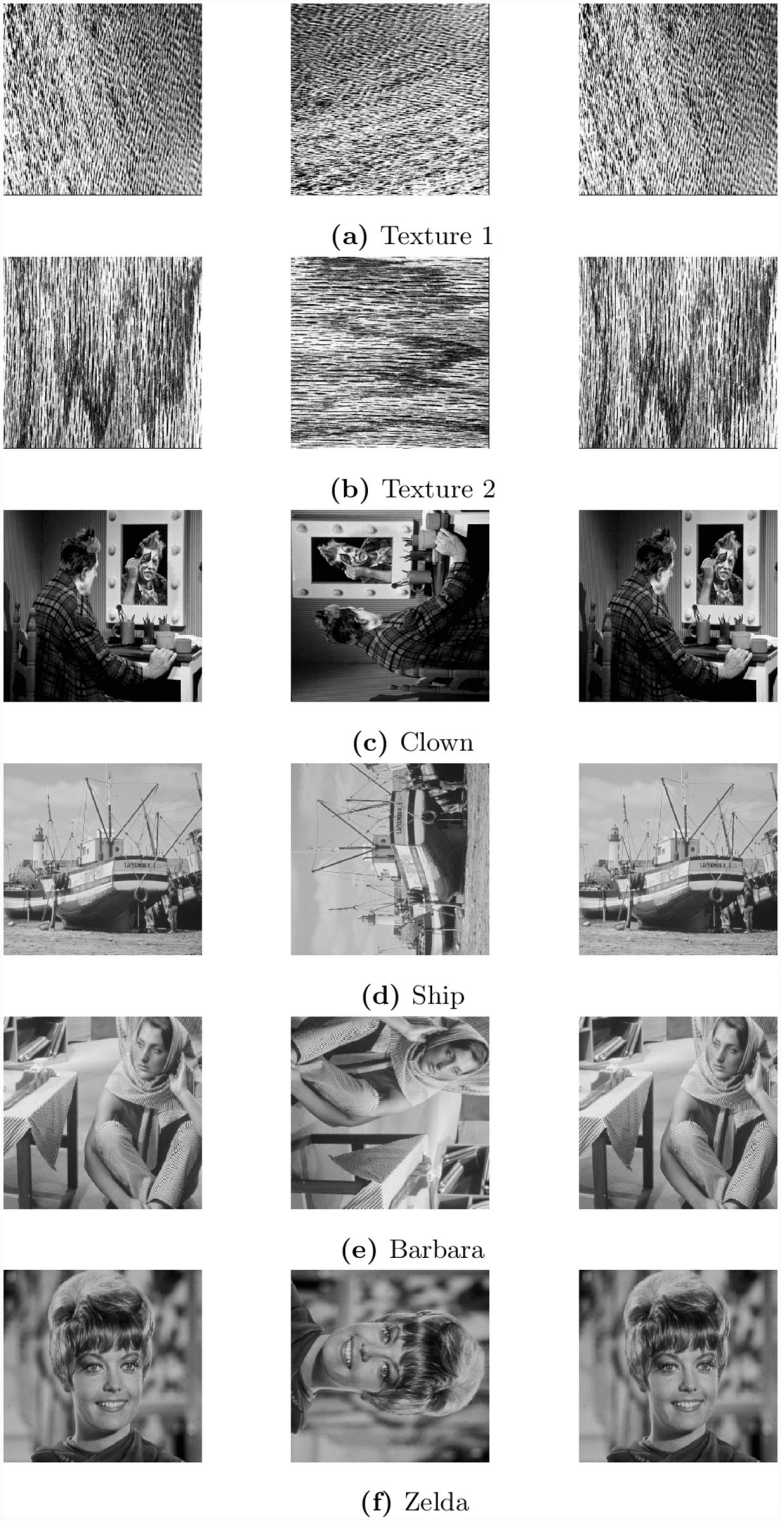
Input images, transformed images and marked images.

**Fig 12:**
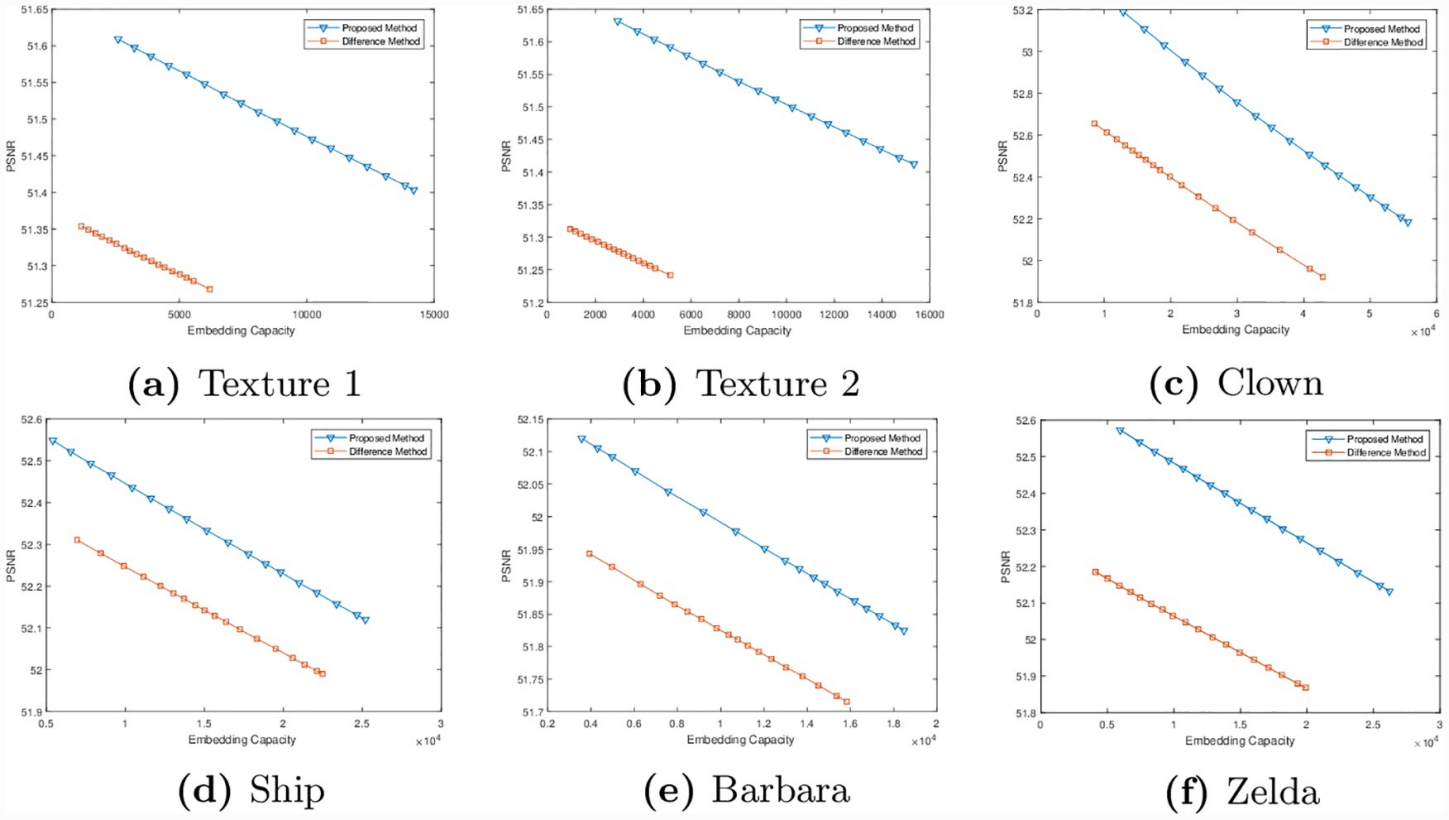
Comparisons of embedding capacity and PSNR in proposed and difference methods.

Note that in all the above cases, we have achieved maximum capacity using column-wise difference. In some cases, using a row-wise difference can create more embedding capacity compared to the one that uses a column-wise difference. This happens when image has high variation along column as compared to rows. Some preliminary work in this regard has already been reported in [42], where both horizontal and vertical directions to embed data are utilized. Here we show that we can achieve higher embedding capacity, if we rearrange the rows of an image. To demonstrate this, we have selected a set of six publicly available images. This set includes the images of textures, clown, Ship, Barbara and Zelda. These images are shown in the left most column of Fig 11. For each of these images, we have performed a 90° rotation and computed the embedding capacity for different values of *δ* based on row-wise difference. Next we have embedded data in each of these images and performed an inverse rotation. The rotated images and the images with embedded data are shown in the middle and the right most columns of Fig 11. Note that except for the case of Barbara and Zelda, the value of *δ* was equal to 1, therefore the images were only rotated clock-wise. For the images of Barbara and Zelda, the images was rotated as well as flipped. This is due to the reason that the value of *δ* for these images was equal to 511. This means that the first column is subtracted from the last one, third from second and so on. In this case, the sequence of indices produced by Algorithm 1 is 1, 512, 511, 510, …, 3, 2. This causes the image to be flipped. To compare the performance of the two methods, we have computed the values of PSNR for both methods and shown in Fig 12.

The proposed method is compared with multilevel histogram modification of difference images by Lin et. al [9], Histogram shifting by Ni et. al. [43], and Histogram Modification of Difference Images by Lee et. al [44]. The maximum capacity of embedding data for all images presented in this section by the proposed method and compared with three state-of-the-art techniques are shown in Table 7. The results demonstrate that the proposed method has significantly achieved higher embedding capacity and PSNR compared to existing difference methods. In checker board the embedding capacity is more than 100%, while in images with pattern the embedding capacity is more than 50% compared to existing difference methods. Our method performs very well when there are regular patterns in the image. So textures and pattern images are giving higher capacity. For irregular images, the capacity may or may not be increased. Results show that the embedding capacity can be improved, if a row-wise difference is used instead of a column-wise difference. It is important to note that we have not only applied row-wise difference, but also rearranged the rows according to the index map that is produced by Algorithm 1.

**Fig 13:**
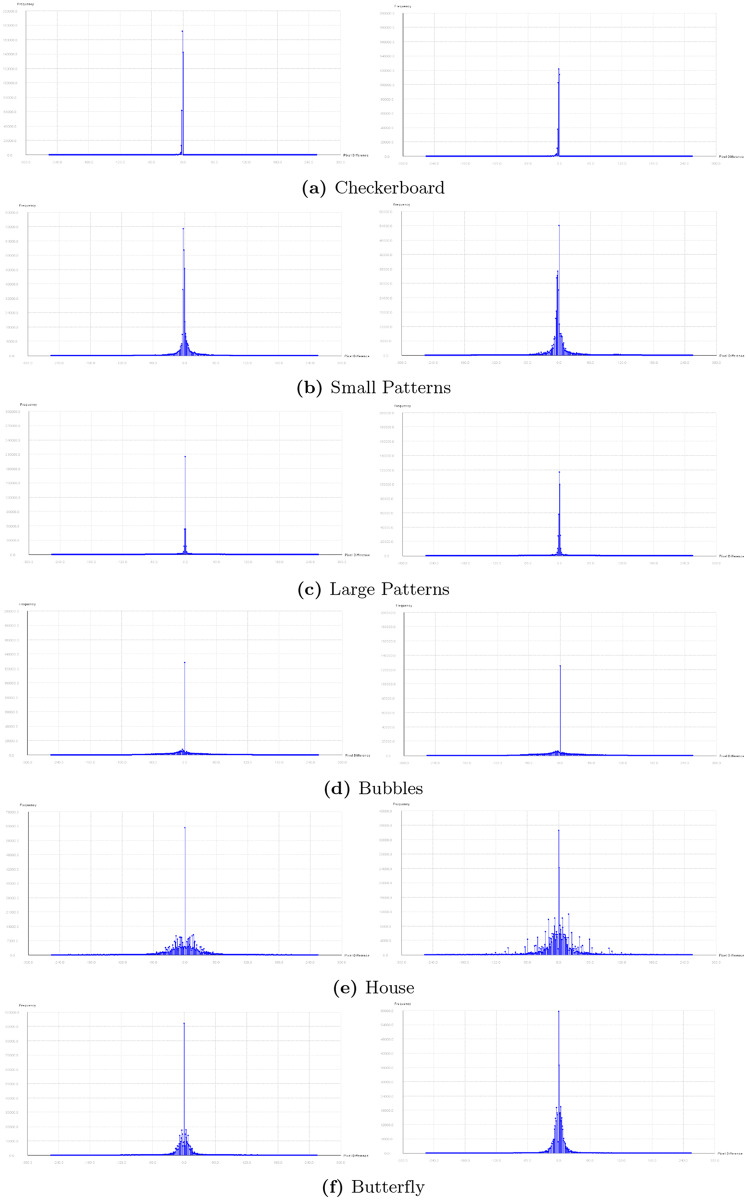
Pixel Difference Histogram (PDH) for input images and marked images (part I).

**Fig 14:**
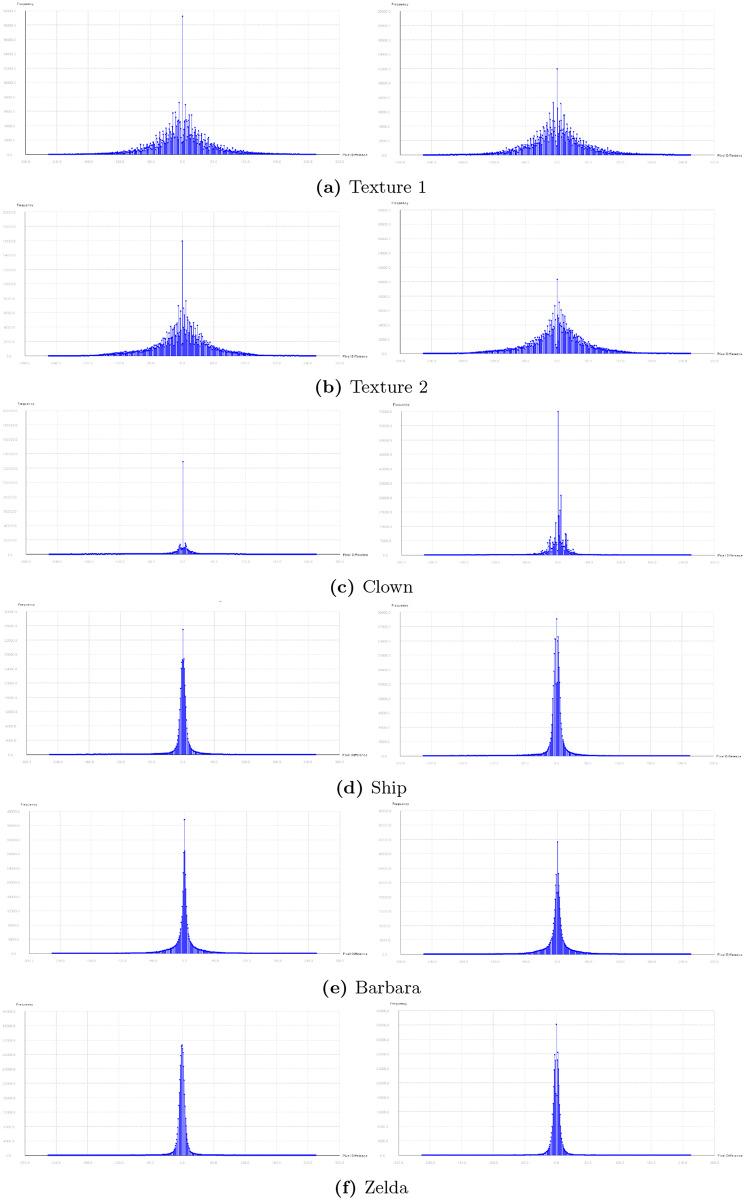
Pixel Difference Histogram (PDH) for input images and marked images (part II).

The Pixel Difference Histogram (PDH) analysis is performed for all images used in this paper and is shown in Figs 13 and 14. The histogram for the input images are very similar to marked images. This steganalysis demonstrates that ability of the proposed algorithm to embed data securely. According to [1] PDH of any original image will be a smooth curve. If the stego/marked-image is more distorted, its PDH curve becomes zig-zag in nature. If the distortion in the stego/marked-image is very less, then its PDH curve looks smooth like that of an original image. The zig-zag nature of PDH curve is known as the step effect. The PDH analysis for the proposed technique demonstrates that the PDH curves of the stego/marked -images do not show any step effects. Therefore, it is concluded that the proposed technique is undetectable by PDH analysis.

Overall the comparison between the proposed framework and the difference based method shows that our method can significantly increase the embedding capacity of images that have regular patterns. This can be verified from the quantitative results shown in Table 7 which shows that for textures the embedding capacity can be increased by more than 50% (and in some cases more than 100%) with better PSNR. Note that since our approach is based on histogram shifting, it suffers from the underflow/overflow problems. This can be avoided using location map [14], which contains the location information of all selected values. This location map is then lossless compressed and embedded into the marked image together with the message. The overflow/underflow problem can be handled in a way similar to that of difference method. A number of techniques that have been proposed to address the problem of overflow/underflow. These techniques can be directly applied to the proposed method.

## 5 Conclusion

We have presented a new method of reversible data hiding in images that achieves higher embedding capacity and image quality. This is done by rearranging the pixels of the image in a way that enlarges the smooth regions of an image, as smoother regions has higher peak values. This can be very effective when the image has repeating patterns with same intensity variations. For texture images, our proposed framework was able to embed more than twice the data that can be embedded using the existing difference based method, while it also improves the visual quality of the marked image. However, the proposed framework can be easily extended to any other reversible data hiding techniques that exploit the smooth region of an image to increase their embedding capacity. We have also used two different directions for embedding the data i.e. column-wise and row-wise transformation, and have demonstrated that, in some cases, the embedding capacity can also be increased by rearranging the rows of an image.
